# Measuring Kinematic Response to Perturbed Locomotion in Young Adults

**DOI:** 10.3390/s22020672

**Published:** 2022-01-16

**Authors:** Juri Taborri, Alessandro Santuz, Leon Brüll, Adamantios Arampatzis, Stefano Rossi

**Affiliations:** 1 Department of Economics, Engineering, Society and Business Organization (DEIM), University of Tuscia, 01100 Viterbo, Italy; juri.taborri@unitus.it; 2 Department of Training and Movement Sciences, Humboldt-Universität zu Berlin, 10115 Berlin, Germany; alessandro.santuz@hu-berlin.de (A.S.); bruell@nar.uni-heidelberg.de (L.B.); a.arampatzis@hu-berlin.de (A.A.); 3 Berlin School of Movement Science, Humboldt-Universität zu Berlin, 10115 Berlin, Germany; 4 Network Aging Research, Heidelberg University, 69117 Heidelberg, Germany

**Keywords:** perturbed locomotion, fall risk, inertial sensors, gait analysis, coordination

## Abstract

Daily life activities often require humans to perform locomotion in challenging scenarios. In this context, this study aimed at investigating the effects induced by anterior-posterior (AP) and medio-lateral (ML) perturbations on walking. Through this aim, the experimental protocol involved 12 participants who performed three tasks on a treadmill consisting of one unperturbed and two perturbed walking tests. Inertial measurement units were used to gather lower limb kinematics. Parameters related to joint angles, as the range of motion (ROM) and its variability (CoV), as well as the inter-joint coordination in terms of continuous relative phase (CRP) were computed. The AP perturbation seemed to be more challenging causing differences with respect to normal walking in both the variability of the ROM and the CRP amplitude and variability. As ML, only the ankle showed different behavior in terms of joint angle and CRP variability. In both tasks, a shortening of the stance was found. The findings should be considered when implementing perturbed rehabilitative protocols for falling reduction.

## 1. Introduction

Humans have to constantly deal with the challenge to adapt their gait to several environmental conditions; thus, one of the main roles of the central nervous system (CNS) is to assure the maintenance of dynamic stability during locomotion on different types of surfaces [1]. Dynamic stability is defined as the ability to control the body’s center of mass within a moving base of support [2]. An incorrect stability leads to increased risk of falls, which represent a major public worldwide health issue [3]. It was demonstrated that falls mainly occur if the normal gait is disturbed by external factors, such as slips, trips, and collisions, as well as arising from volitional movement, such as turning and bending [4]. The ability to correctly respond to a balance perturbation determines whether or not a fall occurs, and it is shown that this ability can be deteriorated as a consequence of CNS changes and/or muscle properties [5,6]. In this context, perturbation-based gait paradigms have reached greater popularity for the measurement of dysfunctions, diseases, or injuries [7,8,9]. Based on the tested population, perturbations of different natures have been used, ranging from cognitive [10] to visual [11] and physical [12]. At the same time, several variables have been considered as sensitive for the quantification of human responses to gait perturbation; among other, spatio-temporal parameters [13], dynamic stability [13,14,15,16], muscular activity [12,17,18], kinematics [14,16,18], and inter-joint coordination [3,19] have been the most adopted ones.

As concerns spatio-temporal parameters, Madehkhaksar et al. [13] evaluated the effects of sudden, unexpected mechanical perturbation in medio-lateral (ML) and anterior-posterior (AP) directions during treadmill walking in 10 healthy young adults. They found that perturbed locomotion is associated with a shorter stride length, a wider step width and a higher cadence especially when considering ML perturbations. In addition, a greater stride-to-stride variability was found for perturbation-based trials. Such higher variability might be used as an indicator of instability and fall risk. As concerns dynamic stability, Roeles et al. [15] evaluated the effects on ML and AP margins of stability following (1) belt acceleration and deceleration; (2) visual and auditory perturbation in AP and ML directions; and (3) ipsi- and contra-lateral sway. Among others, contra-lateral sway and deceleration perturbations showed different margins of stability with an increase of the area up to 4 and 6 times in ML and AP, respectively. The test was conducted with nine young and nine older healthy adults and obtained similar behavior between the two examined cohorts. Differently, no effects on ML margins of stability were found when young adults reacted to unexpected mechanical perturbation of the supporting base when walking [13]. Considering the muscle activity, Santuz et al. [12], by applying the muscle synergy theory, demonstrated that humans are able to modify their motor control strategies, especially in terms of timing, when walking in unsteady conditions.

Moving to kinematics, the analysis of trunk and upper body segments was deeply conducted in previous studies. As an example, Rum and colleagues [18] quantified the trunk kinematic differences between young and older healthy adults following unexpected waist lateral perturbation during walking. A reduction in trunk movement was found as a significant effect of the aging, underlying a more rigid behavior of the upper body in older adults when they are asked to respond to unexpected perturbation. Finally, the inter-joint coordination has been depicted as an indication having implications for falls in elderly [20]. Forty-six older and younger adults were enrolled in the experimental procedure conducted by Ippersiel et al. [3], who asked participants to perform walking trials on irregular surfaces that randomly tilted in AP and ML directions. A greater variability of inter-joint coordination was observed on uneven walkways with the differences accentuated in older adults especially during early stance phase and mid-swing.

Focusing on this literature overview, it is clear that the analysis of all the cited variables can lead to the identification of useful information to better understand the mechanism of the Central Nervous System (CNS) in controlling perturbed locomotion. To the best of the authors’ knowledge, no studies have explored the effects induced by perturbation on lower limb kinematics in terms of joint angle variations, as well as all the previous studies applying unexpected perturbations to understand human strategies in balance responses. However, it is important to analyze the changes in lower limb joint angles, which represent the most widespread analyzed parameters when performing gait analysis, as well as understanding the reactive response to expected perturbations. In addition, lower limb joints are the most important ones when considering the response of a subject after perturbations of the support base in order to avoid falls; in fact, it is well known that ankle- and/or hip-based strategies are mandatory for maintaining equilibrium [21]. As a consequence, the evaluation of responses to expected perturbations can allow obtaining further information on CNS behavior considering that there are still two open, opposing interpretations of movement changes. In fact, it was shown that the movement changes may be considered not only as incorrect patterns due to the perturbations, but also as exploratory behavior to avoid falls [22]. In this context, this study aimed at measuring the effects induced by expected perturbations in AP and ML directions on treadmill-based gaits of young healthy adults in terms of lower limb joint angles and inter-joint coordination. 

Due to the expected nature of the perturbations, such analysis permitted us to evaluate the modification of the CNS behavior focusing only on the perturbation rather than on other external factors that could influence the gait patterns in case of unexpected perturbations. In addition, humans daily face expected perturbation of their walking patterns, such as walking on uneven ground, ascending or descending stairs, etc. Consequently, the results of this paper can point to important features to consider when implementing gait perturbation paradigms for rehabilitation purpose, i.e., reducing falls among the elderly. 

## 2. Materials and Methods

### 2.1. Participants

Twelve young, healthy participants (four males, age: 26 ± 3 years, height: 170.8 ± 4.7 cm, body mass: 64.9 ± 9.6 kg) were recruited in the experimental protocol. The dominant leg, which was identified by asking participants to kick a ball [23], was right for all the involved participants. Participants were excluded if they had known neuromuscular and vestibular pathologies or, more in general, any disease that affects the walking and balance ability. Each participant was informed of the aims and the experimental procedures and invited to sign an informed consent. All the procedures were in accordance with the Helsinki Declaration.

This study was approved and reviewed by the Ethics Committees of the Heidelberg University (AZ Schw 2018 1/2).

### 2.2. Instrumentation

The perturbed-locomotion protocol was performed by using a treadmill (BalanceTutor™, MediTouch LTD, 15 Netanya, Israel, Movie S1) that was able to provide sudden external perturbations in both the anterior-posterior (AP) and medio-lateral (ML) directions. AP perturbation was achieved by providing a rapid acceleration of the treadmill belt, whereas a sudden displacement of the belt-supporting platform was performed to provide ML perturbation. The kinematics of the lower limb was gathered through commercially available inertial measurement units (IMUs) provided by Xsens Technologies (Enschede, The Netherland). Each sensor was equipped with a triaxial linear accelerometer, triaxial gyroscopes, and a triaxial magnetometer able to respectively acquire the linear acceleration, the angular velocity, and the magnetic field of the body segment it was attached to. The acquisition frequency of IMUs ranged from 40 Hz to 120 Hz. In this study, the acquisition frequency was set to 60 Hz, in accordance with previous studies on gait analysis [24,25]; in addition, the selected frame rate was more than five times higher than both the gait frequency and the frequency of the perturbations, assuring an adequate acquisition without any aliasing effects.

### 2.3. Experimental Protocol

**Figure 1:**
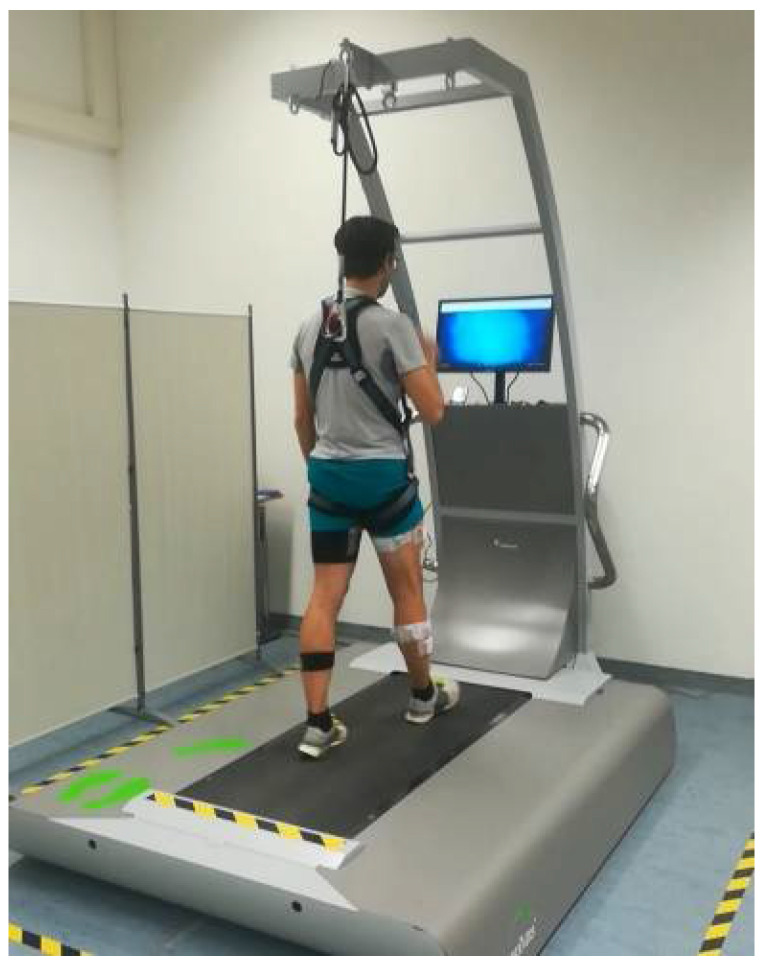
Example of a participant placed on the treadmill with the harness worn and the sensors positioned on the body segments of interest. The sensor on the pelvis was placed under the t-shirt.

Before the experimental session, each participant was sensorized with seven IMUs placed on the pelvis, right and left thighs, right and left shanks, and right and left feet. The placement was performed by the same operator using ad hoc elastic belts that limited the movement artifacts during the execution of the motor tasks. After the sensorization phase, each participant was asked to perform two static tasks; specifically he/she had to maintain a standing upright position and a sitting position for at least 5 seconds each in order to acquire the data mandatory for the functional calibration of the sensors [26]. Successively, each participant was positioned in the center of the treadmill and his/her safety was assured by using a harness attached on the top of the treadmill. Figure 1 reports the placement of a participant on the treadmill with the sensors attached on the body segments and the harness worn. Each participant was asked to wear his/her preferred comfortable shoes.

Before starting with the motion tasks, each participant performed several strides in order to familiarize with both the treadmill speed and the presence of sensors to avoid gait pattern alterations. The familiarization period was stopped when the participants felt her/himself comfortable with the treadmill speed and lasted approximatively 3 min per each participant. No data were acquired during the familiarization phase.

The experimental protocol consisted of three walking tasks. During the first task, hereinafter named as Normal Walking (NW), each participant was asked to walk on the treadmill for 6 minutes. The speed of the treadmill was equal for all the participants and set at 4.3 km/h. The value of the walking speed was in line with average walking speed of pedestrians [27]. Data gathered during NW were used to analyze the gait pattern of each participant in unperturbed condition. 

The second task, hereinafter named as Expected perturbation in Anterior-Posterior (E_AP_), consisted of 3 minutes of normal walking interspersed with three expected perturbations in the anterior-posterior direction. The perturbation consisted of a sudden acceleration of the treadmill belt in the anterior-posterior direction, set at 0.5 m/s^2^ for a duration of 0.5 s. Similarly, the third task, hereinafter named as Expected perturbation in Medio-Lateral (E_ML_), consisted of 3 minutes of normal walking interspersed with three expected perturbations in the medio-lateral direction. In this case, the perturbation, which lasted 0.5 s, consisted of a rapid displacement of the treadmill base in the medial direction with the values of displacement and acceleration equal to 16 cm and 0.18 m/s^2^, respectively. All the perturbation parameters were selected during a pilot study conducted on three participants in order to optimize the perturbation amplitude avoiding the subject’s fall. 

In both AP and ML, each participant was instructed to normally walk and react to the sudden perturbation, fixing a monitor in front of him/her in which the countdown and the direction of the perturbation were reported. Thus, it was clear that participants were aware about strides with and without perturbations. The perturbations started during the mid-stance of the right foot, as in [28,29]. The heel strike was recognized by the central unit and the sensor embedded in the treadmill. It is worth noting that no constraints were imposed on the participant’s reaction; however, the trial was repeated if the participant performed a step outside the treadmill belt to recover balance after the perturbation. Data gathered during both AP and ML were used to analyze how the cohort of healthy young participants reacted to sudden, expected perturbation in different directions and then compared with the ones gathered during the NW task.

The entire protocol lasted approximatively 30 min per each participant, including the sensorization phase, and all the enrolled participants were able to complete the required tasks. No repetition of the task was necessary in E_AP_, whereas three of 14 participants had to repeat ML since he/she reacted with a step outside the treadmill.

### 2.4. Data Processing and Analysis

For all the motion tasks, the angles of hip, knee, and ankle joints were extracted by applying an ad hoc implemented biomechanical model and the functional calibration proposed in [26]. Considering the sagittal plane as the one mainly involved in the walking, only angles related to this plane were elaborated. In addition, only data related to the right lower limb were analyzed, considering that the perturbations were provided on the right side.

As concerns NW, angle time series were partitioned into each gait cycle, which was defined as the time between two consecutive heel strikes of the same side. The partitioning was performed by applying an already validated threshold-based algorithm based on shank angular velocity, as reported in [30]. In particular, the algorithm was based on the search of a relative maximum and the minimum immediately before that corresponded to the heel strike. After the partitioning, each stride was normalized at 100 samples. Before the computation of the synthetic indices, the strides related to the first and the last minute of the task were discarded from the successive analyses in order to avoid bias due to acceleration and deceleration phases. Successively, the mean angle curve $\vartheta_{m}^{\mathrm{NW}}$ was computed by averaging across all the strides for each participant and each joint. Then, the Range of Motion (ROM) was computed as the difference between the maximum and minimum angle value within the considered stride, independently per each stride, each joint, and each participant. Finally, the mean value (${\mathrm{mROM}}^{\mathrm{NW}}$) and its standard deviation (${\mathrm{sdROM}}^{\mathrm{NW}}$) were obtained by considering the ROM values across the strides for each joint and each participant. To quantify the gait variability, the Coefficient of Variation (${\mathrm{CoV}}^{\mathrm{NW}}$) was computed as the ratio between ${\mathrm{sdROM}}^{\mathrm{NW}}$ and ${\mathrm{mROM}}^{\mathrm{NW}}$, expressed as percentage. The Higher the value of the CoV, the greater the variability of the kinematics.

As regards the inter-joint coordination, the continuous relative phase (CRP) technique was adopted [31]. The CRP allowed us to quantify the coupling behaviors of two body segments over the entire duration of the motor task [32]; essentially, it represents the phasing relationship between the actions of two segments to perform a specific movement. Hip, knee, and ankle angular velocities were computed as the first derivative of the joint angle curve multiplied for the transformation matrix H, which took into account the selected Euler sequence. Successively, the phase angle of each joint was computed as the inverse tangent of the joint angular velocity $\dot{\vartheta }$ divided by the joint angle curve ϑ at any point time $t_{i}$, as in the equation:

$$\varphi \left(t_{i}\right)=\arctan\left(\frac{\dot{\vartheta }\left(t_{i}\right)}{\vartheta \left(t_{i}\right)}\right)$$

Finally, CRP was computed by subtracting the distal and proximal joint phase angles. CRP values can range between −360° to 360°, with values close to 0 ± 360° indicating that the two segments were moving following an in-phase coupling, whereas −180° and 180° represent an out-phase coupling behavior [33]. CRP was computed for the following joint pairs: (1) hip and knee ${\mathrm{CRP}}_{\mathrm{HK}}^{\mathrm{NW}}$; (2) hip and ankle ${\mathrm{CRP}}_{\mathrm{HA}}^{\mathrm{NW}}$, and (3) knee and ankle ${\mathrm{CRP}}_{\mathrm{KA}}^{\mathrm{NW}}$. This procedure was repeated individually for each stride and each participant.

As synthetic indices, the Mean Absolute Relative Phase (MARP) and Deviation Phase (DP) were used. Firstly, we computed the ensemble CRP curve by averaging the CRP across the stride of the same walking condition for each participant. Then, the MARP was determined by averaging the absolute value of the ensemble curve points for the overall duration of the task T, as in the following equation [34]:

$$\mathrm{MARP}=\frac{\sum_{i=1}^{T}\left|{\mathrm{CRP}}_{i}\right|}{T}$$

Instead, DP was evaluated by averaging the standard deviations of the ensemble CRP points (SD_i_) for the overall duration of the task T, as in the following equation:

$$\mathrm{DP}=\frac{\sum_{i=1}^{T}SD_{i}}{T}$$

DP values close to 0° indicate lower stride-to-stride variability. The DP index allows quantifying the stride-to-stride variability associated with the inter-joint coordination and it is typically used as a measure of the stability during locomotion. It is clear how both indices, MARP and DP, were computed for all the three above-mentioned joint pairs.

By moving to E_AP_ and E_ML_, only the perturbed strides were taken into account. The identification of the perturbed strides was conducted by using the output of the control system of the treadmill. In fact, it was possible to extract the instant of the start and the stop of each perturbation. These time instants were used to divide the stride of the entire trial and select only the ones associated with a perturbation provided by the treadmill. This approach allowed us to avoid using as normal walking strides the ones before and after perturbations since each participant was aware on the perturbation timing and she/he could modify the gait patterns to easily react to the perturbations. A post-processing technique was applied to assure the synchronization between the data recorded by the IMUs and the outputs provided by the treadmill. Specifically, when starting the experimental task, the treadmill belt moved backward at a constant speed and the participant was asked to follow the belt without starting to walk. Such movement can be easily detected by analysing the linear acceleration provided by the IMU place on the foot, which was post-processed by an ad-hoc algorithm to synchronize the signals. After the segmentation, the same parameters of the normal walking were computed. For sake of clarity, it should be noted that all the unperturbed strides during E_AP_ and E_ML_ tasks were discarded from the successive analyses.

**Figure 2:**
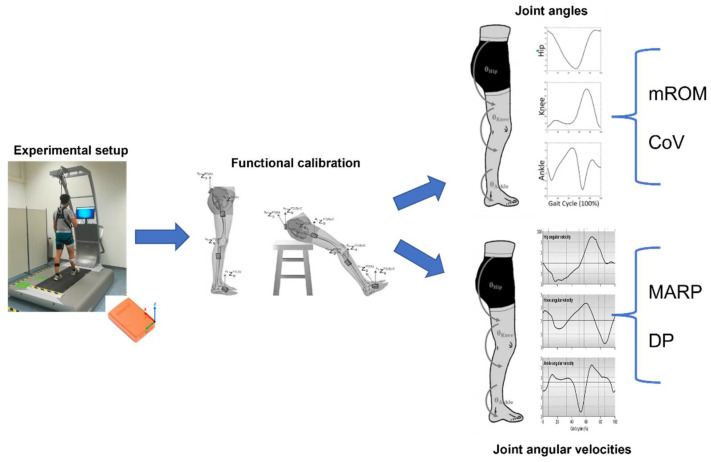
Schematic summary of experimental setup, data processing, and analysis.

Figure 2 shows a schematic summary of the experimental setup, data processing, and analysis.

### 2.5. Statistical Analysis

Considering the mean joint angle curve $\vartheta_{m}^{}$, a statistical parametric mapping (SPM) one-way repeated measurement ANOVA was performed to examine whether the three motion tasks, i.e., NW, E_AP,_ and E_ML_, differed significantly from each other. The test was performed individually per each joint. The same test was also used to evaluate the effects of the different motion tasks on CRP mean curves. For each SPM ANOVA, a statistical parametric map SPM{F} was created by calculating the conventional univariate F-Statistic at each point of the gait curve [35]. If SPM{F} crossed the threshold line corresponding to a confidence level equal to 0.95, post-hoc SPM{t} maps were calculated for comparing each pair of independent variables. When the SPM{t} map crossed the critical threshold, a significant difference was found between the examined pair of motion tasks.

Conversely, when analyzing mROM, CoV, MARP, and DP, firstly all data were tested for normality through the Shapiro–Wilk test, and successive one-way repeated measure ANOVA tests were performed independently per each parameter and each joint by considering the three motion tasks as independent variables. When the assumption of sphericity was violated, the Greenhouse–Geisser correction was considered. In the case of statistical differences, a Bonferroni test for multiple comparisons was applied. For all the performed statistical tests, a significance level equal to 0.05 was considered.

## 3. Results

**Figure 3:**
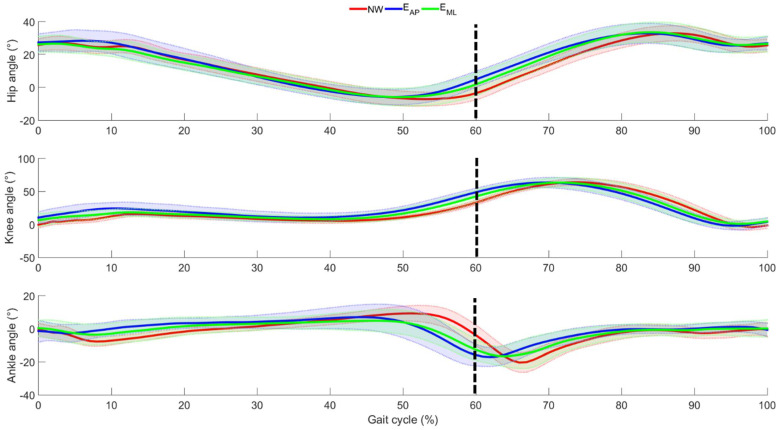
Mean (bold line) and standard deviation (shaded area) of the lower limb joint angles as averaged across participants. Red, blue, and green are used to identify the normal walking, the perturbed locomotion in anterior-posterior direction, and the perturbed locomotion in medio-lateral direction, respectively. The dotted, black line represents the transition between stance and swing phases in NW.

The mean joint angles and the relative standard deviations across all participants are depicted in Figure 3 considering all the three motion tasks.

**Figure 4:**
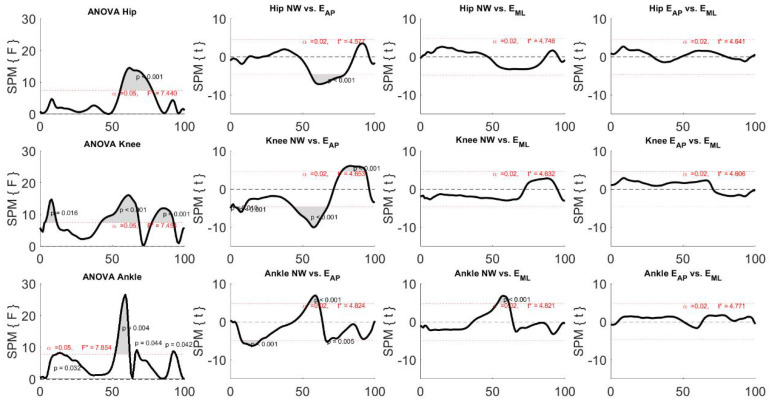
Results of the one-way repeated measurement ANOVA SPM and related post-hoc analysis for the joint angle curves. Black, bold lines indicate the statistical maps and the dotted, red lines indicate the critical thresholds, whereas the grey areas indicate the significant difference among independent variables, i.e., the three motion tasks. F* and t* indicate the F-value and t-value for ANOVA and t-tests, respectively.

From Figure 3, it can be observed that the general behavior of the lower limb joints was tendentially kept similar among the three walking conditions. The main difference was observed for the ankle joint due to a decrease of both dorsi and plantarflexion when the locomotion was perturbed both in AP and ML directions. An anticipation of the overall minimum of the ankle angle was also associated with the perturbed locomotion. Generally, such anticipation was observed also for the knee and hip joints, even if the timing difference was found less evident when analyzing the joints distal with respect to the perturbations. This consideration was also confirmed by the SPM analysis conducted on the mean joint angle and reported in Figure 4.

By analyzing Figure 4, it is seen that all the ANOVA tests indicated the presence of statistical differences among the three motion tasks. Specifically, when focusing on the comparison between NW and E_AP,_ all the three examined joints were influenced by the perturbation, especially during the swing phase (after 50% of the gait cycle) and the knee and ankle joints also in the first subphase of the stance period. Furthermore, only the ankle joint between 50% and 60% of the gait cycle showed differences between NW and E_ML_, whereas no difference was found when comparing the two types of perturbed locomotion.

**Figure 5:**
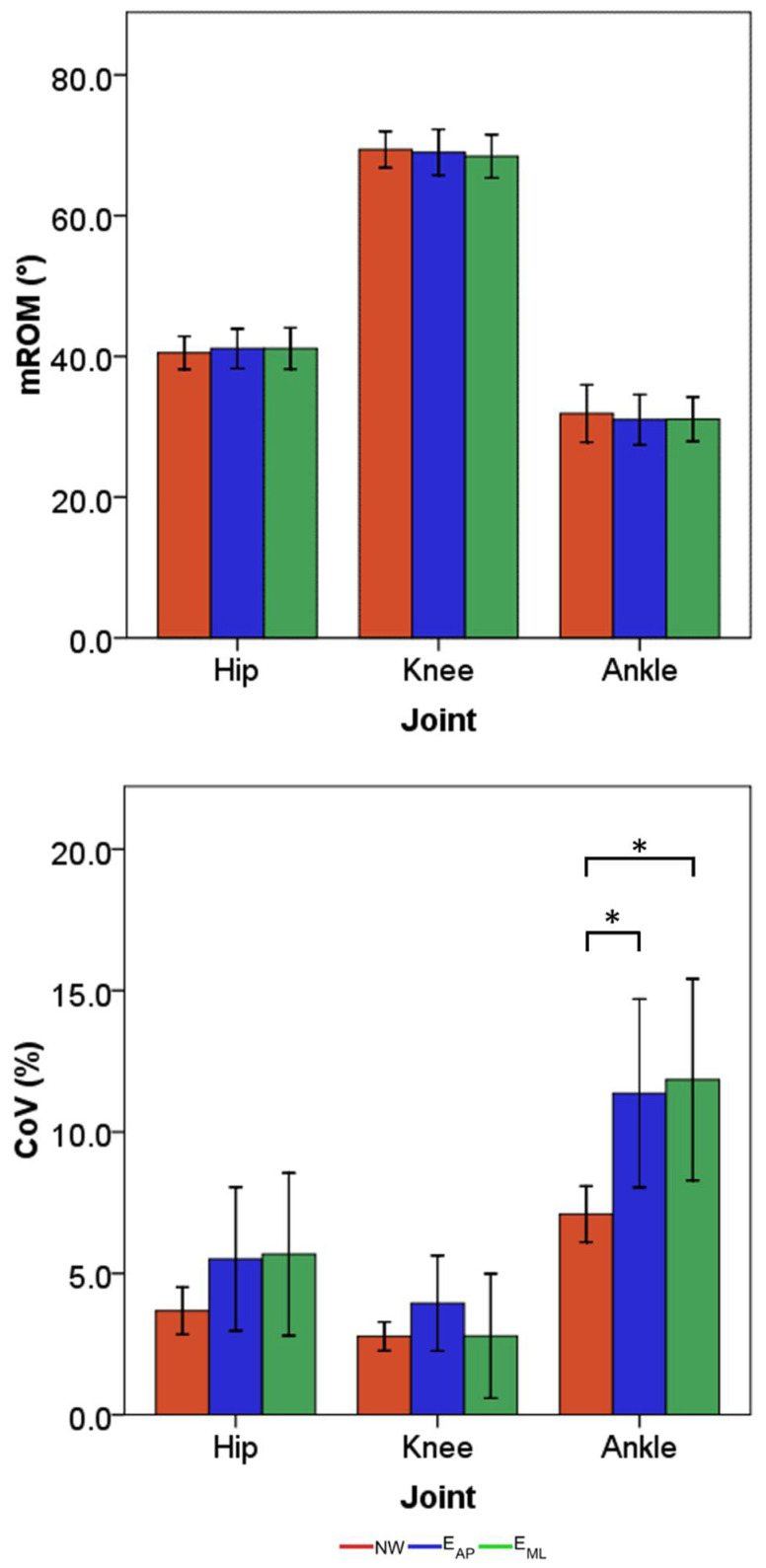
Means and standard deviations of the mROM (**top**) and CoV (**bottom**) for all three motion tasks. Red, blue, and green are used to identify the normal walking, the perturbed locomotion in anterior-posterior direction, and the perturbed locomotion in medio-lateral direction, respectively. * is placed in correspondence to statistical differences among tasks.

Considering the synthetic indices, the mean mROM and the mean CoV across participants with their relative standard deviations are reported in Figure 5. Despite the differences found when comparing the angle curves, the mROM values achieved during all the three tasks did not lead to statistical differences among motion tasks. The same results were also associated with the hip and knee joints when considering the CoV values. Conversely, the variability related to the ankle joint was found to be significantly higher in the two perturbed locomotion tasks with respect to normal walking, up to a variability greater than 10.0% for both tasks. The standard deviation of the variability for all the examined joints was substantially higher when looking at the results related to the perturbed locomotion tasks.

**Figure 6:**
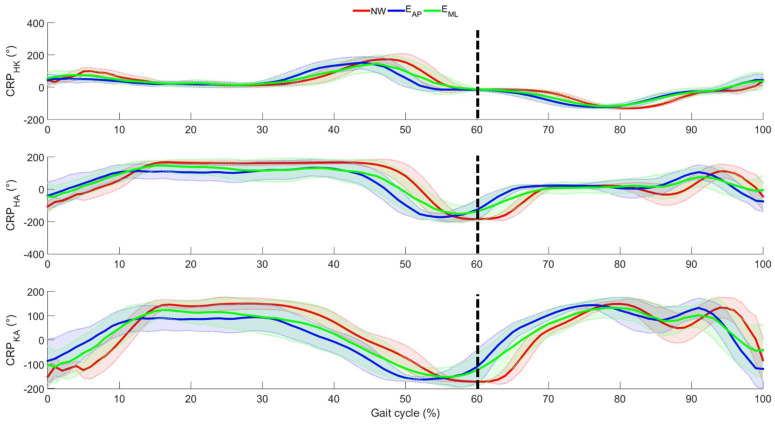
Mean (bold line) and standard deviation (shaded area) of the lower limb joint CRP as averaged across participants. Red, blue, and green are used to identify the normal walking, the perturbed locomotion in anterior-posterior direction, and the perturbed locomotion in medio-lateral direction, respectively. Dotted, black line represents the transition between stance and swing phases in NW.

**Figure 7:**
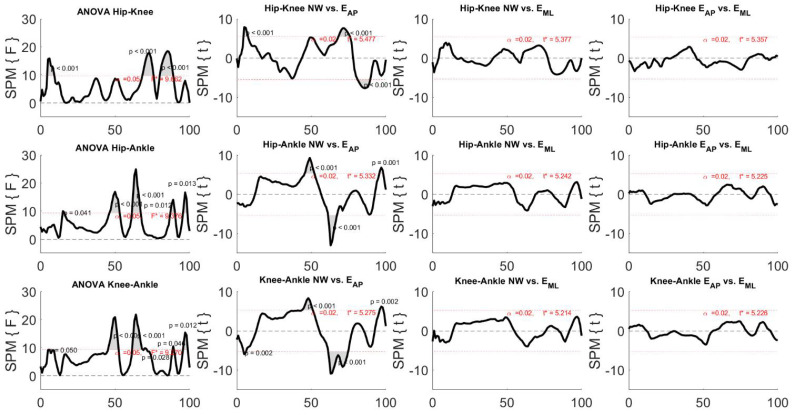
Results of the one-way repeated measurement ANOVA SPM and related post-hoc analysis for CRP curves. Black, bold lines indicate the statistical maps, the dotted, red lines indicate the critical threshold, and the grey areas indicate the significant difference among independent variables, i.e., the three motion tasks. F* and t* indicate the F-value and t-value for ANOVA and t-tests, respectively.

By moving the inter-joint coordination analysis, the mean CRP curves and related standard deviations are reported in Figure 6 and the results of the one-way repeated measurement ANOVA SPM is reported in Figure 7.

It was found that ANOVA tests always revealed statistical differences. In particular, the differences were found only when comparing NW and E_AP_, whereas no difference was noticed in the other compared pairs. More specifically, the differences were observed (1) at the first phase of the stance both for the couple hip-knee and the couple knee-ankle; (2) at the last phase of the stance both for the couple hip-ankle and the couple knee-ankle; (3) immediately after the start of the swing phase, i.e., approximatively 60% of the gait cycle, in all the examined inter-joint couples; (4) at the midswing only in the couple hip-knee; and (5) immediately before the heel strike both for the couple hip-ankle and the couple knee-ankle.

**Figure 8:**
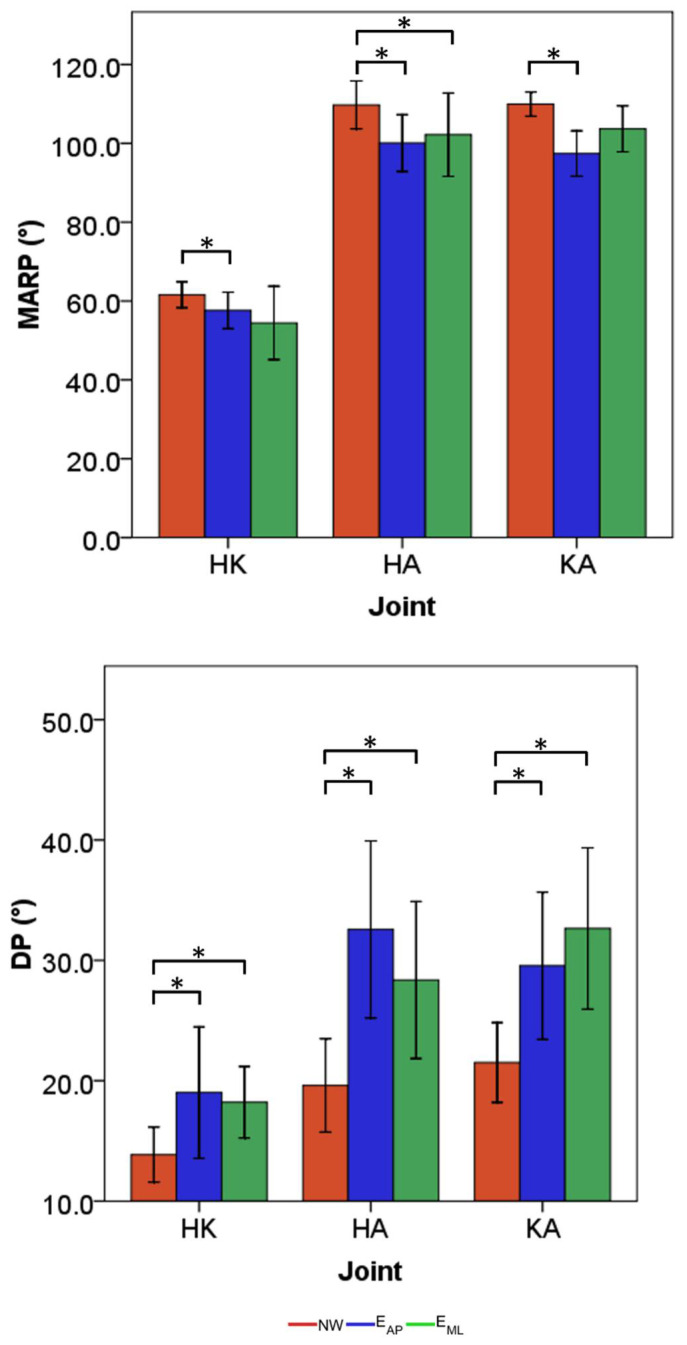
Means and standard deviations of the MARP (**top**) and DP (**bottom**) for all the three motion tasks. Red, blue, and green are used to identify the normal walking, the perturbed locomotion in anterior-posterior direction, and the perturbed locomotion in medio-lateral direction, respectively. * is placed in correspondence to statistical differences among tasks.

The results obtained for the synthetic indices computed on the CRP curves and the relative statistical findings are reported in Figure 8.

By considering the MARP, statistical differences were found between NW and E_AP_ for all the three examined joint pairs (*p*-values ranged from 0.01 to 0.03), whereas the index related to E_ML_ was not different from NW in the couple hip-knee. Generally, the indices computed for the normal walking tasks achieved the greatest values of MARP with 62°, 110°, and 110° for the pairs HK, HA, and KA, respectively. As far as DP, the two motion tasks in which a participant was subjected to external perturbations were associated with values statistically greater than the ones related to the normal walking tasks (*p*-values ranged from <0.01 to 0.04). The lowest value of DP was related to the HK in normal walking, equal to 14°, whereas the greatest was associated with KA in E_ML_ task, equal to 33°.

## 4. Discussion

Through the aim to assess the effects induced by external expected perturbations to level locomotion in healthy subjects, we analyzed lower limb joint angles and inter-joint coordination parameters in a cohort of 12 participants when performing three motions, i.e., normal walking, walking with perturbations in the AP direction, and walking with perturbations in the ML direction.

### 4.1. Effects of Perturbation on Lower Limb Joint Angles

The findings revealed that the curves related to all the three examined joints were characterized by a topological similarity among the unperturbed and the perturbed locomotion. Focusing on perturbed locomotion, it was clear how the differences with respect to normal walking were mainly due to a different distribution of the timing events within the stride. In fact, a shortening of the stance period was observed in both AP and ML data by considering the early ankle plantarflexion and hip-knee flexion immediately before the transition between the stance and the swing phases. The SPM outcomes also confirmed this consideration since the differences were not related to all the gait cycles but only at one specific time interval, i.e., the transition between stance and swing phases, allowing assessing the presence of a phase-dependent compensatory responses [36]. These results are in line with previous studies that demonstrated that the shortening of the stance duration is one of the main strategies adopted by healthy humans when recovering the stability after a perturbation of the normal locomotion [36,37]. In addition, the early heel-off and the reduced flexion of the stance limb were typical mechanisms adopted to increase the toe clearance of the contralateral leg, useful to avoid trip and/or fall at the successive step of the swing leg [38]. Even though a direct comparison with previous studies could not be conducted due to the absence in the literature of kinematic analysis related to a similar protocol, we can affirm that the physiological kinematic patterns highlighted by the outcomes are consistent with the findings of studies based on the analysis of muscular activity in the same challenging scenarios, especially considering the anticipation found for the lower limb muscle activation [39].

Despite the differences during the gait cycle, absence of differences in terms of range of motion was observed. However, such a finding confirmed that the range of motion can be considered a kinematic parameter invariant across several locomotion-related tasks, as also reported for other parameters related to kinematics and muscle activity [40]. Thus, we concluded that the recovery of stability after a perturbation of the locomotion was achieved by changing the stride time in terms of percentage between stance and swing and not by modifying the sagittal angular excursion of the lower limb joints. Thus, the modification of the stride time seemed to be the approach selected by young adults to minimize the variation of the ROM. Despite the invariance of the ROM, a greater stride-to-stride variability was found in both perturbed tasks, previously highlighted by the CoV. This outcome is also in line with previous studies that affirmed that the presence of an external perturbation always led to a reduction of the gait speed, which was inversely proportional to the gait variability [41]. 

Finally, since the absence of statistical differences between the two perturbed tasks was mainly due to the high inter-subject variability related to ML, some qualitative speculations on different strategies can be noted. The differences with respect to normal walking observed for all the examined joints when analyzing AP data allowed us to affirm that the entire biomechanical chain is influenced by the anterior-posterior perturbation and, consequently, we can speculate that the ankle is not able to absorb the disturbance. Such a result, combined with the already discussed shortening of the stance, suggests that participants are forced to change gait from walking to running in order to avoid trips and/or falls. This aspect is well recognized as one of the main strategies adopted by the central nervous system when an anterior-posterior perturbation is provided [42]. In addition, the reduction of the plantarflexion at the loading response is a consequence of a decrease in the tibialis anterior activity, also typical of running [43]. Conversely, when looking at the curves related to the ML, only the ankle joint showed differences with respect to normal walking, allowing us to affirm that the ankle is able to absorb the medially directed perturbation without transmitting it to the remaining biomechanical chain. This consideration, combined with the shortening of the stance always present also in ML tasks, can be ascribed to the stepping strategy, which is considered the most adopted reaction to mediolateral perturbation [44]. This is also confirmed by the cross-over step of the contralateral leg that often occurred during the experimental protocol when ML perturbations were provided. In fact, the cross-over step is a typical mechanism of the stepping strategy, as demonstrated in [45].

By summarizing, the main effects caused by expected anterior-posterior and medio-lateral perturbations are the shortening of the stance and an increment of the stride-to-stride variability. Such effects are probably due to two distinct strategies that are a walking–running transition in case of anterior-posterior perturbations and stepping strategy when inward perturbations are provided.

### 4.2. Effects of Perturbation on Lower Limb Inter-Joint Coordination

As for the joint angle curves, the trend of the continuous relative phase is in accordance with the ones reported for normal walking of healthy subjects in previous studies [3,31,33]. CRP curves related to the three examined motion tasks are topologically similar; differences were observed for all the three joint pairs in the case of anterior-posterior perturbations. The differences in the early stance can be ascribed to a different activation of the anterior tibialis and, consequently, of the ankle and knee movements, mandatory for foot flattening, which can be influenced by support base disturbance [46]. Furthermore, the presence of differences in the transition between stance and swing is a consequence of the gait pattern associated with the hip and knee joints that have an important role for the toe clearance, which is affected by the anterior-posterior perturbation [31]. As for the differences in terminal swing, they were ascribed to a different preparation to the successive strike of the perturbed limb [3].

Focusing on the synthetic indices, both perturbed tasks are generally characterized by an increment of the in-phase coupling of the lower limb joint, attesting from the significant reduction of the CRP amplitude (MARP). Contrary to what is thought, a more in-phase coordination does not mean a better working of the central nervous system but only a reduction of independent action associated with each segment, which is the physiological behavior that guarantees the prevention of falls [3]. The increment of in-phase pattern can be, instead, ascribed to a more cautious gait pattern, typically adopted by humans to avoid trips and/or falls; in fact, it is shown that the central nervous system works in this direction to uphold a stable position [47]. Furthermore, an always greater variability was found in perturbed tasks. Such increased variability can be justified by a greater challenge for the neuromuscular system in adapting the gait pattern to external disturbance [48]. In addition, a greater variability has already been associated with a poor dynamic balance of the stance limb [49] and a strategy that leads to augmenting the toe clearance [50], adopted by humans to avoid falls. As a conclusion, low variability of gait patterns, and specifically of the inter-joint coordination, has been often used as indirect indices to asses stability during locomotion [33]. The findings related to the amplitude and variability of CRP are in line with the ones reported in [3] when analyzing inter-joint coordination of human locomotion over irregular terrains; similarly, the reduction of CRP amplitude was observed in perturbed locomotion characterized by obstacle crossing [34] and slowing forced at slower speeds [51].

These findings, combined with the ones related to the joint angles, should be taken into account for implementing perturbation paradigm programs addressed to increase the adaptation of a subject to challenging walking scenarios [40], using the proposed indices to monitor the progress and the effect of the programs.

## 5. Conclusions

Understanding how the central nervous system modifies the gait patterns in response to external perturbation of locomotion can lead to useful information for implementing perturbation rehabilitative programs, as well designing control systems for exoskeletons to use in daily life. The findings of the present study reveal that different physiological gait patterns in terms of joint angles and inter-joint coordination are found when anterior-posterior and inward expected perturbations are provided. Both perturbations lead to a shortening of the stance, an increment of the gait variability, and modifications of amplitude and variability of the inter-joint coordination. No differences were statistically appreciable between both pattern and synthetic indices associated with the two perturbed tasks; however, two different balance recovery strategies were adopted, which were walking–running transition in the case of anterior-posterior perturbations and a stepping strategy when locomotion was perturbed in an inward direction. Further studies should apply the same methodology in order to investigate the effects of aging; as well, the analysis of out-of-sagittal planes should be performed to fully characterize the compensatory mechanism adopted by CNS.

## Data Availability

All raw data are available by contacting the corresponding author.
